# SRBreak: A Read-Depth and Split-Read Framework to Identify Breakpoints of Different Events Inside Simple Copy-Number Variable Regions

**DOI:** 10.3389/fgene.2016.00160

**Published:** 2016-09-15

**Authors:** Hoang T. Nguyen, James Boocock, Tony R. Merriman, Michael A. Black

**Affiliations:** ^1^Department of Biochemistry, University of OtagoDunedin, New Zealand; ^2^Virtual Institute of Statistical GeneticsDunedin, New Zealand; ^3^Department of Psychiatry, Mount Sinai School of Medicine, New YorkNY, USA; ^4^Department of Mathematics, Cao Thang College of TechnologyHo Chi Minh City, Vietnam

**Keywords:** read depth, copy number variant (CNV), split read, breakpoint cluster region, structural variation (SV)

## Abstract

Copy-number variation (CNV) has been associated with increased risk of complex diseases. High-throughput sequencing (HTS) technologies facilitate the detection of copy-number variable regions (CNVRs) and their breakpoints. This helps in understanding genome structure as well as their evolution process. Various approaches have been proposed for detecting CNV breakpoints, but currently it is still challenging for tools based on a single analysis method to identify breakpoints of CNVs. It has been shown, however, that pipelines which integrate multiple approaches are able to report more reliable breakpoints. Here, based on HTS data, we have developed a pipeline to identify approximate breakpoints (±10 bp) relating to different ancestral events within a specific CNVR. The pipeline combines read-depth and split-read information to infer breakpoints, using information from multiple samples to allow an imputation approach to be taken. The main steps involve using a normal mixture model to cluster samples into different groups, followed by simple kernel-based approaches to maximize information obtained from read-depth and split-read approaches, after which common breakpoints of groups are inferred. The pipeline uses split-read information directly from CIGAR strings of BAM files, without using a re-alignment step. On simulated data sets, it was able to report breakpoints for very low-coverage samples including those for which only single-end reads were available. When applied to three loci from existing human resequencing data sets (NEGR1, LCE3, IRGM) the pipeline obtained good concordance with results from the 1000 Genomes Project (92, 100, and 82%, respectively). The package is available at https://github.com/hoangtn/SRBreak, and also as a docker-based application at https://registry.hub.docker.com/u/hoangtn/srbreak/.

## Introduction

Copy number variation (CNV) has been associated with increased risk of complex diseases such as austim, HIV, Crohn’s disease, rheumatoid arthritis, epilepsy, bipolar disorder, Alzheimer’s disease, and obesity (Gonzalez et al., 2005; McCarroll et al., 2008; Bentley et al., 2009; McKinney et al., 2010; Chung et al., 2014; Falchi et al., 2014; Hooli et al., 2014; Olson et al., 2014; Green et al., 2016). In addition, CNV at the CCL3L1 locus has also been associated with selective adaptation (Gonzalez et al., 2005; Perry et al., 2007; Hardwick et al., 2011, 2014). Such CNV-disease relationships, however, are difficult to detect and replicate for a number of reasons (He et al., 2009; Shrestha et al., 2010; Carpenter et al., 2011; Nordang et al., 2012; Aklillu et al., 2013). One reason for this relates to technical limitations – gold standard methods for measuring CNV, like the paralog ratio test, are less amenable to higher throughput genotyping, meaning that inferior methods such as quantitative PCR are more often employed (McKinney and Merriman, 2012). A second reason is that, owing to the often recurrent nature of recombination events leading to common CNV, surrogate tag single nucleotide polymorphisms (SNPs) that are easy to genotype often do not exist. Given also the recurrent recombination events, a third reason is that the breakpoints of copy-number variable regions (CNVRs) have not been precisely identified. The occurrence of multiple independent ancestral copy number changes at a locus can lead to different sub-regions having the same copy number, but different breakpoints, which could result in different pathways of disease (e.g., some breakpoints could disrupt the function of a gene more than others). Therefore, precise identification of the breakpoints of duplication or deletion events could enhance our understanding of the exact structure of regions carrying the CN variants, and the subsequent functional impact on biological pathways. Furthermore, these exact breakpoints would be amenable to direct genotyping for surrogate measurement of CNV.

While an appreciable percentage (20–40%) of normal and pathogenic CNVs have been shown to have non-recurrent breakpoints (Conrad et al., 2010; Arlt et al., 2012), CNVs associated with complex diseases are known to have recurrent breakpoints. For example, a common 20-kb deletion upstream of the IRGM gene has been associated with Crohn’s disease (McCarroll et al., 2008), a common 40-kb deletion upstream of the NEGR1 gene has been associated with body mass index (Willer et al., 2009), and a 32-kb deletion around the LCE3C and LCE3B genes has been associated with psoriasis (de Cid et al., 2009). Moreover, at some complex loci, breakpoints have been identified at hotspots of non-allelic homologous recombination, or in concentrated clusters (Lindsay et al., 2006). For instance, a 129-kb polymorphic region on chr12p13.31 associated with rheumatoid arthritis has multiple left breakpoints in a region of 1200 bp, and multiple right breakpoints in a region of 2100 bp (Veal et al., 2014). Similarly, for the FCGR3B gene that is associated with systemic lupus erythematosus (SLE) and rheumatoid arthritis, multiple breakpoints of deletions lie in a 24.5-kb region (Mueller et al., 2013). Independent recurrent deletion events at the same locus were also found in the SIRPB1 gene by Kidd et al. (2008). This largely precludes using a tag SNP approach for detection and genotyping of complex CNV, although this has been demonstrated to be feasible in some populations at the FCGR3 locus (Nguyen et al., 2013).

Using high-throughput sequencing (HTS) data, multiple pipelines have been developed to detect and genotype structural variations (SVs) in the human genome. Frequently, read depth (RD), paired-end mapping (PEM), split-read, local assembly or some combination of two more of these approaches is used to detect SVs. The RD approach is based on read counts aligned to regions of the genome, and uses changes in read depth to identify regions of CNV (Abyzov et al., 2011; Koboldt et al., 2012; Nguyen et al., 2013; Wang et al., 2013). The PEM approach uses the distance and directions of two ends of a DNA segment to infer a SV event. For example, if the mapped distance of two reads is larger/smaller than their expected distance then this indicates a deletion/insertion event (Korbel et al., 2007; Chen et al., 2009; Zeitouni et al., 2010; Rausch et al., 2012; Hart et al., 2013). Reads which are aligned across breakpoints are usually split into separate parts and only some parts are mapped, therefore split read based pipelines use the information from these reads to infer SV events and their breakpoints (Ye et al., 2009; Rausch et al., 2012; Hart et al., 2013; Wu et al., 2013).

Both split-read-based and assembly based approaches can report base-pair resolution of breakpoints (Zhao et al., 2013), therefore integrated pipelines which include these two approaches to obtain high resolution estimation of breakpoints have been developed (Ye et al., 2009; Wong et al., 2010; Wang et al., 2011; Hart et al., 2013). For example, SVMerge (Wong et al., 2010) uses PEM and read-depth methods to detect SVs and then an assembler is used to assemble reads around detected breakpoints into breakpoint-containing contigs, which are then aligned to the reference genome to refine the estimated positions of the breakpoints (Wong et al., 2010). Other tools such as DELLY (Rausch et al., 2012), LUMPY (Layer et al., 2014) and SoftSearch (Hart et al., 2013) combine PEM, read-depth and split-read information to obtain estimates of breakpoint locations.

Here, we introduce an integrated analysis framework (SRBreak) which combines a read-depth-based approach and a split-read-based approach to identify breakpoints for different duplication/deletion events inside a large CNVR. The strength of this pipeline comes through its use of multiple samples in one CNV genotype group to identify common breakpoints for that group. It is able to use both single-end and paired-end reads from HTS data.

## Methods and Data

In this work, both simulated and real data were used to characterize the performance of the SRBreak methodology. We simulated a 1 Mb region containing different duplication and deletion events, and also low coverage data from a full chromosome (chromosome 21). For real data, three human loci with disease-associated CNV were used (NEGR1, LCE3, IRGM).

### Simulated Data

#### Simulation of a 1 Mb Region

A 1 Mb segment on chromosome 1 (chr1:101100001-102100000) was extracted from the human reference genome (version hg19). On this 1 Mb segment, different recombination events including duplications and deletions were simulated. The breakpoints relating to these events are described in **Table 1**. The software package dwgsim^1^ was used to simulate HTS read data from this region with the following parameters: paired reads (inner distance = 500 bp), read lengths = 100 bp, rate of mutations = 0.001; single-end reads, read lengths = 100 bp, rate of mutations = 0.001. BWA-MEM (Li, 2013) was then used to align simulated read data to the reference genome. The coverage used in the simulated data was set from 1 to 15x (sample coverages range from 1 to 15x as in **Table 1**). **Supplementary Figure S1** shows plots of simulated events.

**Table 1:** Breakpoints of simulated samples for single- and paired-end reads.

	Start	End	Length (bp)	Coverage	Sample number
Del	101545220	101630000	84780	1–15x	15
Del	101556000	101576000	20000	1–15x	15
Del	101560000	101565000	5000	1–15x	15
Del	101561000	101562000	1000	1–15x	15
Dup	101555000	101605000	50000	1–15x	15
Dup	101556000	101576000	20000	1–15x	15
Dup	101558000	101568000	10000	1–15x	15
Normal				1–15x	15
Total					120

#### Simulation of a Chromosome

A VCF for chromosome 21 was downloaded from the 1000 Genomes Project ftp://ftp.1000genomes.ebi.ac.uk/vol1/ftp/data/ on 26 January, 2015. A list of all annotated SVs within the chromosome was obtained. From this list, 20 loci with different sizes ranging from ∼1000 to ∼10000 bp were randomly chosen. Five samples with coverage from 1 to 5x for the whole of chromosome 21 with deletions at the 20 loci were simulated. Paired-end reads whose lengths are 100 bp were used in this process. Mappability information was downloaded from http://hgdownload.cse.ucsc.edu/goldenPath/hg19/encodeDCC/wgEncodeMapability/wgEncodeCrgMapabilityAlign100mer.bigWig on 26 May, 2014 (Karolchik et al., 2014). Segmental duplication information was downloaded from http://humanparalogy.gs.washington.edu/build37/data/GRCh37GenomicSuperDup.tab on 26 May, 2014 (Bailey et al., 2001).

#### Human High-Throughput Sequencing Data

Three loci, a region upstream of the NEGR1 gene (NEGR1 locus), a region upstream of the IRGM gene (IRGM locus) and a region including the LCE3B and LCE3C genes (LCE3 locus) were used in this study. Deletions of these loci were reported by Tuzun et al. (2005), and later studies confirmed these results, with improved resolution of breakpoints (Redon et al., 2006; Korbel et al., 2007; McCarroll et al., 2008; Conrad et al., 2010; The 1000 Genomes Project, 2012). At the NEGR1 locus, the breakpoints reported by The 1000 Genomes Project (2012) are nearly identical to those of Conrad et al. (2010). At the LCE3 locus, very similar breakpoints were reported by three studies (Korbel et al., 2007; Conrad et al., 2010; The 1000 Genomes Project, 2012). At the IRGM locus, McCarroll et al. (2008) reported breakpoints using microarray data, and validated the precision of the breakpoints using PCR assays. Although the initial results from The 1000 Genomes Project (2012) did not include the breakpoints of McCarroll et al. (2008), the updated data sets from the 1000 Genomes Project (6 November, 2014)^2^ confirmed the breakpoints of McCarroll et al. (2008). Based on the agreement between these studies, here the published results of The 1000 Genomes Project (2012) and the results of McCarroll et al. (2008) were used to test the breakpoint detection pipelines.

The breakpoints of the NEGR1, LCE3 and IRGM loci are chr1:72,766,323-72,811,840, chr1:152,555,542-152,587,742 (The 1000 Genomes Project, 2012) and chr5:150,203,163-150,223,264 (McCarroll et al., 2008), respectively. Using the breakpoint information, Tabix (Li, 2011) was used to download VCF files (Danecek et al., 2011) from http://ftp.1000genomes.ebi.ac.uk/vol1/ftp/phase1/analysis_results/integrated_call_sets/ and to extract sequence from the three loci (January 7, 2015). There were 1092 samples included in these VCF files. Within these 1 Mb regions, there were other deletions (≥1000 bp, minor allele frequencies > 0.0025) which had been reported by the 1000 Genomes Project. We also obtained breakpoints of these deletions and used them to compare all pipelines. The breakpoints associated with these deletions are presented in **Supplementary Table S1**.

On January 7, 2015, a list of the 2,535 latest samples of the 1000 Genomes Project was obtained from the link: ftp://ftp.1000genomes.ebi.ac.uk/vol1/ftp/data/. Four populations, CEU and YRI, CHB and JPT, for which CN at the three loci has previously been measured experimentally (Redon et al., 2006; McCarroll et al., 2008; Conrad et al., 2010) were chosen to assess the performance of SRBreak and other methods from the literature. There were 415 CEU and YRI, CHB and JPT samples. A 1 Mb region around the three loci was extracted: chr1:72,200,001-73,200,000, chr1:152,000,001-153,000,000, and chr5:149,500,000-150,500,000 for the NEGR1, LCE3 and the IRGM loci, respectively. Samtools (Li et al., 2009) was used to generate BAM files (Li et al., 2009) for the 415 samples around the two 1 Mb regions.

### SRBreak Pipeline

**FIGURE 1:**
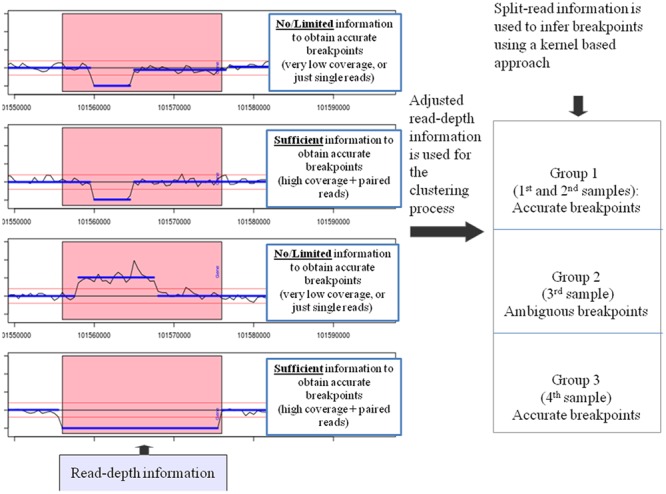
**SRBreak: read-depth information is adjusted for GC-content and mappability biases.** Adjusted read-depth information is used to obtain CNVRs using thresholds*;* red horizontal lines (left panel). Pink rectangles describe a large CNVR including three different events. The first two samples depict a deletion event with the same breakpoints in both samples; the third sample is a duplication event and the fourth sample is a deletion event. Next, for each CNVR, a normal mixture model is used to cluster samples into different groups and read-depth based boundaries are inferred. After that, paired-end information (if it is available) is used to refine the boundaries for each group. Finally, split-read information around these boundaries is used to infer breakpoints based on a kernel-based approach. For each plot on the left panel, the x-axis describes genome coordinates while the y-axis depicts standardized read counts (RCs).

An overview of the SRBreak (“Split-read and Read-depth based Breakpoints”) analysis pipeline is presented in **Figure 1**. The following description provides a brief overview of the SRBreak methodology, as applied to the data sets analyzed here. Duplicate reads were removed from the BAM files using Samtools (Li et al., 2009) and read counts were generated in non-overlapping windows across the 1 Mb regions of interest. These reads were then corrected for GC bias and mappability bias using the method of Yoon et al. (2009). For each sample, read counts in each window were divided by the median read counts across windows to transform all samples to the same standardized scale. These transformed read counts were then decreased by 1, so that a value of zero would correspond to a diploid region. In the package, there is an option in which users can manually standardize across windows and then standardize across samples as in our previous work (Nguyen et al., 2013) and use these values as input into the pipeline. However, this option should only be used if users know that the total sizes of duplications/deletions are smaller than the total sizes of normal regions [if the total sizes of duplications/deletions are larger than the total sizes of normal regions, then duplications/deletions can appear to be “normal” regions (i.e., CN = 2) after the standardization process].

**FIGURE 2:**
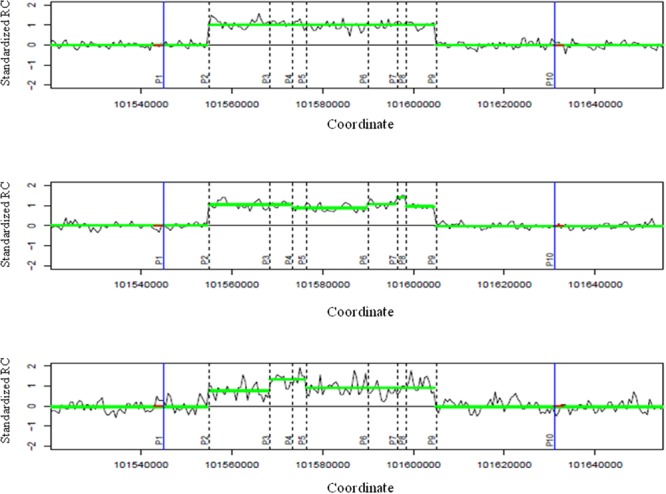
**Identifying breakpoints using read-depth information for a group.** Three illustrative plots describe the segmentation results of three samples around a CNVR. The two blue vertical lines are the two approximate boundaries of the CNVR. These two boundaries encompass nine sub-regions for the group with 10 positions [P1 to P10, *N_breaks_* = 10, *x_k_* (*k* = 1.10)] are the coordinates of these 10 points, each *x_k_* of each sample has a weight *w_k_.* For example, the top sample has *w_k_* = 0 if *k* = 1, 3- > 8, 10 because there are no differences in segmentation results of two sub-regions around these breaks, and *w_k_* ≠ 0 if *k* = 2, 9 because there are differences in segmentation results of the two sub-regions at the second and ninth breaks). These sub-regions are non-overlapping regions which were counted by extracting all segmentation results across all samples. To calculate kernel-based scores for positions, zero values (red color) were added at the approximate boundaries. P2 and P9 would have the highest kernel-based scores and are the two breakpoints of the group. For each plot, the x-axis describes genome coordinates while the y-axis depicts standardized read counts (RCs).

Standardized read counts from all samples were segmented into different groups having similar read-count information using the CNVrd2 package (Nguyen et al., 2014). Segmentation results were then used to identify sub-regions of constant CN. Sub-regions, as described in Nguyen et al. (2014) and in **Figure 2**, are non-overlapping regions which are inferred from the segmentation results across the samples used in one analysis. If the segmentation result for a sub-region was larger/smaller than a threshold, then the sub-region was called as a duplication/deletion. Therefore, for each sample, each sub-region described one of three possibilities: duplication, deletion, or no variation. To choose a suitable threshold for duplication and deletion events, we tested different thresholds, with the goal of obtaining a small threshold that was balanced between true positive and false discovery rates (FDRs). In an ideal situation, thresholds larger than 0.5 and smaller than -0.5 would be suitable for calling the two events respectively. However, because of the noise of read counts, reads from duplication regions could be mapped to other regions, and reads from other regions could be mapped to deletion regions. In addition, windows which flank breakpoints could have standardized read counts higher/lower than 0.5/-0.5 because they included a duplication/deletion and a normal region. Therefore, it is likely that this approach would miss regions of duplication or deletion if such high thresholds were used. To avoid this, different thresholds (0.5, 0.35, 0.25, 0.2) were tested to call a sub-region as a CNV region. Consecutive duplication/deletion sub-regions were merged to form CNVRs. All sub-regions were used in the merging process including regions with very high standardized read-count signals. The notation used in the pipeline is presented in **Supplementary Table S2A**.

For each CNVR, the following steps were used:

#### Step 1: Identify Groups with Different CN Using Read-Depth Information

At each CNVR, a *M × N* matrix of segmentation values was obtained, where *M* was the number of samples and *N* was the number of sub-regions. Detailed information of sub-regions are described in **Figure 2**. A multivariate normal mixture model was used to cluster samples into different groups using the package mclust (Fraley and Raftery, 2009). Different model types in the package were tested for the clustering process, with *modelNames = EII* (*spherical, equal volume*) found to be more accurate than other models on simulated data (**Supplementary Table S2B**). Therefore, this model was chosen to obtain groups having similar segmentation results across the CNVR. The Bayesian Information Criterion (Schwarz, 1978) was used to choose the number of components for the normal mixture model. The mclust package automatically tested nine different models (component numbers were from 1 to 9) and chose the one having the highest BIC value as defined by Fraley and Raftery (1998).

#### Step 2: Identify Common Breakpoints for Each Group Using Read-Depth Information

For each group including *M_g_* individuals, a *M_g_ × N* sub-matrix was extracted from the larger *M × N* matrix (Step 1). Breakpoints for the group were inferred from this sub-matrix. The sub-matrix was extended by adding one sub-region to each side of the CNVRs, and all segmentation results from all *M_g_* samples in the extended sub-region were set to zero. This was based on the hypothesis that neighboring regions of the CNVR would exhibit minimal variability in segmentation results. In an ideal situation, we would see the same breakpoints of read-depth methods across samples. A simple approach to obtain breakpoints would be to aggregate breakpoint signals across samples for each position, and then choose the two positions (i.e., start and end of deletion or duplication) having the highest signals. However, to generalize all situations in which breakpoints of samples could scatter around common breakpoints because of noise from the segmentation process, we used a strategy to aggregate all information from the position of interest, and other nearby positions, with closer positions given greater weight. To achieve this, a kernel-based approach was used to obtain breakpoints. A normal (Gaussian) kernel was used in this study because it was simple and met this weighting principle. There were multiple samples, therefore, at each position we aggregated breakpoint information across samples. If the data were not noisy then the simple approach described above could achieve similar results as this kernel-based approach. The following formula was used to calculate the score for each position between two sub-regions (**Figure 2**).

(1) $$scoreP_{j}^{R}=\sum_{i=1}^{M_{g}}\sum_{k=1}^{N_{break}}w_{k}^{R}\times f(x_{k})$$

where *f* is a normal kernel,

$$f(x_{k})\;=\;\frac{1}{\sigma^{R}\sqrt{2\pi }}e^{-\frac{1}{2}{\left(\frac{x_{k}-\mu_{j}^{R}}{\sigma^{R}}\right)}^{2}}$$

with $\mu_{j}^{R}$ representing the coordinate of the *jth* position, *σ^R^* is the standard deviation used in the kernel, $w_{k}^{R}$ is the weight of a position that was calculated as the absolute subtraction of two segmentation results of two sub-regions nearby, N_break_ is the number of boundary points of sub-regions, *x*_k_ values are coordinates boundary points (see detailed information in **Figure 2**). *σ^R^* = a third of window size was used in this study (window size/3).

Based on (1), the base positions corresponding to CNV breakpoints would have higher scores than other positions. In order to more precisely obtain the positions of the breakpoints, resampling *N_r_* times with replacement was performed for each of the *M_g_* samples, and the two positions having the highest scores were recorded each time. The leftmost (i.e., 5′) of the two positions was called the left breakpoint and the other was called the right breakpoint. This resulted in *N_r_* pairs of left and right breakpoints after *N_r_* repeats of the resampling process. The two final breakpoints were generated by calculating the medians of the left and right breakpoints. These were named *MedLeft* and *MedRight*.

#### Step 3: Refine Predicted Breakpoints Using Split-Read Information (**Supplementary Figure S2**)

If reads are aligned across breakpoints then some parts of them cannot be mapped the first time (for some packages, they can be remapped). These parts are denoted by the ‘S’ character in the CIGAR strings of these reads. For each group, split-read positions (*N_splitP_*) from all samples in the group were pooled to begin the process of refining breakpoint predictions. Scores for these positions (*scoreP^S^*) were calculated using the following formula,

(2) $$scoreP_{j}^{S}=\sum_{k=1}^{N_{splitP}}w_{k}^{S}\times f(x_{k}).$$

The weight $w_{k}^{R}$ in the equation is given by the number of reads that were split at the *k^th^* position. *x_k_* values are the coordinates of split-read positions. Based on (2), positions that had more split reads, or positions whose neighbors had more split reads, would have higher scores than others. Finally, left and right breakpoints were the positions having the highest score in the region *MedLeft/MedRight* ±*𝜀_Open_* respectively. In this study *𝜀_Open_* = *window size*.

**FIGURE 3:**
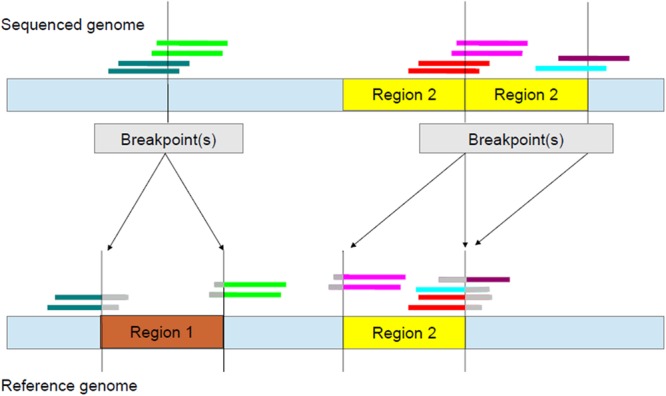
**Identifying breakpoints using split-read information.** In the reference genome (bottom), there is one copy of each of Region 1 and Region 2. However, in the sequenced genome (top), Region 1 is deleted and Region 2 is duplicated. For deletions, CIGAR strings of left breakpoints have a string of bases containing the S character on the right while those of right breakpoints have a string of S characters on the left (**Supplementary Table S3**). The S characters are represented by gray.

As can be seen in **Figure 3** and **Supplementary Table S3**, the CIGAR strings of left and right breakpoints relating to a deleted event are different. Therefore, for deletions, *w_k_* values of left (right) breakpoints were based on CIGAR strings of the Left (Right) CN Group as described in **Supplementary Table S3**.

For each sample, the final result included: CN status (duplication, deletion, or no variation) and predicted break-points.

### Other Packages

Four other packages which use split-read information to infer breakpoints were also used in this study. Pindel (Ye et al., 2009), DELLY (Rausch et al., 2012), SoftSearch (Hart et al., 2013), and MATCHCLIP (Wu et al., 2013). Pindel uses paired-end information as its main approach. It first identifies paired reads for which only one end is mapped and then it breaks unmapped ends into small parts. Next, these small parts are remapped to the reference genome to infer SV events using the information from the mapped ends. DELLY also uses paired-end information to obtain putative boundaries of SVs, and then infers breakpoints by aligning unmapped or partially mapped reads around the SVs to the reference genome. In contrast, both SoftSearch and MATCHCLIP do not use the realignment step, they use split-read information directly from BAM files to infer breakpoints (Hart et al., 2013; Wu et al., 2013). SoftSearch and MATCHCLIP are single-sample based tools (one sample in each analysis) while Pindel and DELLY can use single or multiple samples. These tools have been shown to be effective on real and simulated data sets. Pindel was one of the first tools using a split-read approach to detect different ranges of deletion events (up to 10 kb) with single base resolution (Ye et al., 2009). It is effective on both simulated and real data sets (Ye et al., 2009), and has been used in The 1000 Genomes Project (2010, 2012). DELLY showed better performance than other tools (including Pindel) on simulated data sets, and when applied to the 1000 Genomes Project data DELLY was able to recover 76% of SVs that were detected when 19 bioinformatics tools used by the 1000 Genomes Project were combined (Rausch et al., 2012). The SoftSearch methodology was included in a comparison with other methods applied to simulated data sets, and was found to report more exact breakpoints than other tools (Hart et al., 2013). On a non-simulated high-coverage (2,000x) data set, this package was also able to identify a 71 bp tandem duplication at the BRCA2 locus while two other tools did not report this variant (Hart et al., 2013). The MATCHCLIP software was found to provide a similar level of performance when compared to four other tools, which also include the split-read approach; CREST (Wang et al., 2011), PRISM (Jiang et al., 2012), PINDEL and DELLY, in simulation studies, despite the fact it is a purely split-read based method (Wu et al., 2013). We also included CNVnator (Abyzov et al., 2011), a CNV detection tool based solely on read-depth, in our comparisons.

In the analyses presented here, MATCHCLIP (matchclips1) was run with default parameters. For Pindel (version 0.2.4t), the parameter M was set to 1 (-M 1) and for SoftSearch (version 2.4), the two parameters r and m were also set to 1 (-r 1 -m 1) in order for the packages to call events which have ≥ 1 supporting reads. DELLY (version 0.5.9) was run with default parameters. These parameters were set in order for these packages to be able to obtain all information of very low-coverage samples. MATCHCLIP and SoftSearch were run for single samples while Pindel and DELLY were run for all pooled samples. CNVnator (version 0.3.2) was run with default parameters.

### Performance Assessment

To determine the performance of each method, the following measurements were used: true positive rate (TPR), FDR, and concordance rate (CR). These are defined as:

TPR = (Number of true predicted breakpoints)/(all breakpoints)
FDR = (Falsely predicted breakpoints)/(all predicted breakpoints)
CR = Number of SVs called by a pipeline/Number of true SVs

“True” breakpoints of SVs were defined as those that were previously detected by McCarroll et al. (2008) and The 1000 Genomes Project (2012).

As reported by previous studies (Conrad et al., 2010; Ottaviani et al., 2014) and Abyzov et al. (2015), sequence micro-insertions can occur at deletion breakpoints. These can range from 1 to 96 bp and the majority of the lengths of these sequences are less than 10 bp (Abyzov et al., 2015). Based on this, a predicted breakpoint was considered to be a “true predicted breakpoint” if:

|predicted breakpoint – actual breakpoint|≤ 10 bp

Based on the results of the breakpoint detection, a sample was considered to be a “true predicted sample” if:

1. It had normal CN and it was predicted to have normal CN.
2. It was duplicated/deleted and it was predicted to be a duplicated/deleted sample with two correct predicted breakpoints.

To demonstrate the characteristics of SRBreak, the pipeline was first applied to all 120 simulated samples with different read-depth based window sizes, after which a window size was chosen. Resampling, without replacement, was then performed 250 times for different sample sizes (100, 50, 25, 10, 5) and the pipeline was run on these re-sampled sample sets with the most reliable window size chosen from the results of 120 samples. TPR and FDR were calculated for all analyses. In addition, the first, second and third quantiles of the three measurements across 250 resampled data sets were calculated for each of the simulations.

For the real data sets, we compared the results from this study with those of The 1000 Genomes Project (2012) and (for *IRGM*) McCarroll et al. (2008). A SV which was called by tools used in this work was considered as identical to that of McCarroll et al. (2008) or The 1000 Genomes Project (2012) if its breakpoints were within 10 bp of the two previous results.

To better understand the influence of mapping qualities on the performance of SRBreak on real data sets, we also tested SRBreak with mapping-quality information. We then removed reads having mapping qualities lower than a threshold. GC-corrected read counts were adjusted for mappability bias using the same method as the GC-correction step. After that, SRBreak was used to detect breakpoints for the three loci NEGR1, LCE3 and IRGM. This work was carried out three times for three different mapping-quality thresholds: 1, 10, and 20.

## Results

The SRBreak, Pindel, DELLY, SoftSearch, and MATCHCLIP pipelines were used to analyze the simulated data sets. Performance statistics were then calculated to assess the ability of each approach to detect the simulated breakpoints. To calculate TPRs and FDRs, we focused on results flanking the simulated CNVR. The simulated CNVR was chr1:101,545,220-101,630,000 (**Table 1**), therefore, the results are reported on the region (101,545,220 – 5,000, 101,630,000 + 5,000). To choose a suitable window based on read depth for the analysis, different windows were used on simulated data sets of 120 samples. **Supplementary Table S4** shows distances between predicted breakpoints and real breakpoints for all pipelines on 120 samples. To assess the ability of SRBreak on a whole chromosome, five samples with 20 deletions on the whole of chromosome 21 were simulated.

### Performance of SRBreak on 1 Mb Simulation

Different windows and different thresholds, along with different models in the mclust package for the clustering process were tested. The EII model (spherical, equal volume) achieved greater accuracy than other models; therefore it was used in this study (**Supplementary Table S2B**). We aimed to obtain a small threshold which could call all CNVRs as well as balancing between TPRs and FDRs. The threshold >0.25 for duplications and < -0.25 for deletions satisfied this criterion (**Table 2**), therefore we used these thresholds in the current study. In addition, we also used a threshold σ equal to a third of the window size in this study because it was found to be more reliable across windows compared with other thresholds and the simple approach that did not use the kernel-based method approach (**Supplementary Table S2C**).

**Table 2:** Results of SRBreak on simulated data sets, using different window sizes and different thresholds to call CNVRs.

	Threshold	Window = 50		Window = 100		Window = 250		Window = 500		Window = 1000	
		TPR	FDR	TPR	FDR	TPR	FDR	TPR	FDR	TPR	FDR
**Paired end**											
	0.50	0.71	0.10	0.94	0.02	0.96	0.01	0.98	0.00	0.82	0.04
	0.35	0.80	0.11	0.91	0.04	0.96	0.01	0.98	0.00	0.82	0.06
	0.25	0.80	0.11	0.94	0.01	0.96	0.01	0.98	0.00	0.82	0.06
	0.20	0.80	0.12	0.94	0.01	0.96	0.01	0.97	0.01	0.81	0.07
**Single end**											
	0.50	0.58	0.10	0.94	0.02	0.97	0.00	0.97	0.00	0.86	0.00
	0.35	0.58	0.10	0.95	0.01	0.97	0.00	0.97	0.00	0.86	0.00
	0.25	0.58	0.10	0.87	0.01	0.97	0.00	0.97	0.00	0.86	0.00
	0.20	0.58	0.10	0.85	0.00	0.97	0.00	0.97	0.00	0.86	0.00

#### Paired-End Reads

High TPRs, ≥0.96, were seen for window sizes of 500 and 250 bp (**Table 2**). TPRs steadily decreased when windows were smaller (e.g., 100 and 50 bp), and these measurements were also lower (0.84) for larger windows (1000 bp). FDRs for window sizes of 500, 250, and 100 (≤0.01) were lower than those of window sizes of 50 and 1000 ≥ 0.03.

#### Single-End Reads

Similar to paired-end based results, high TPRs (≥0.97) were seen for window sizes of 500 and 250 bp (**Table 2**). TPRs steadily decreased when windows were smaller (e.g., 100 and 50 bp), and these measurements were also lower (0.86) when window sizes of 1000 bp were used. Interestingly, FDRs were zero for almost all window sizes.

Manual inspection of the SRBreak output revealed that inexact results were observed for window sizes of 1000 bp for both single and paired-end reads (chr1:101,561,000-101562000), with SRBreak not reporting breakpoints for very low coverage samples (1–2x) for this CN event. The best results were seen for window sizes of 500 bp (**Supplementary Table S5**). Based on these results, a window size of 500 bp was used for all of the subsequent analyses.

For a window size of 1000 bp, the TPR of single-end results was slightly higher than that of paired-end results. One possibility for this result was that background noise from the read-depth signals could slightly affect read counts for each window, thus impacting the results of the segmentation and clustering processes inside SRBreak. To test this, we ran paired-end data with 200 different starting positions from 101,100,001 to 101,100,996, and calculated three percentiles (25, 50, and 75%) of TPRs. These values were 0.83, 0.85, and 0.87, respectively, indicating that the results can be affected by read-count noise.

### Impact of Sample Size and Coverage on SRBreak Performance

Results in the previous section were based on a relatively large sample size (120). A resampling process was carried out to allow performance to be calculated across a range of sample sizes. Thresholds and other steps were the same as those implemented in the original analysis of the 120 simulated samples. For each sample size, we randomly sampled 250 times from the 120 samples.

**FIGURE 4:**
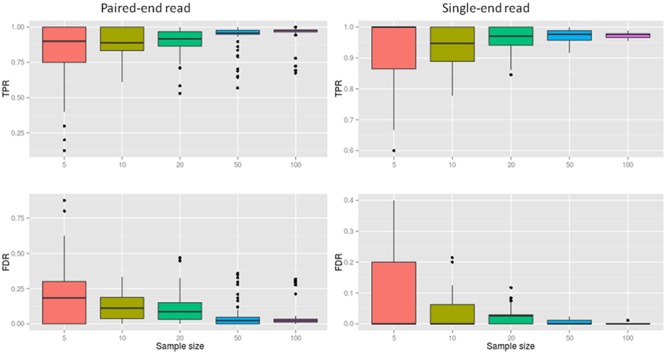
**The performance of SRBreak on simulated data for different sample sizes.** For each sample size, 250 resampling processes were executed.

SRBreak had good performance for large sample sizes, but exhibited low performance when sample size was low. The TPR medians were ≥0.96 with sample sizes ≥50 (**Figure 4**; **Table 3**), but these numbers dropped to 0.89 for sample sizes of 10. Median FDRs were low for all sample sizes of ≥50 (≤0.2). For sample sizes of ≤50, even though all FDR medians were below 0.2, there were multiple occasions when this measurement was higher than 0.2, especially for a sample size of five (**Figure 4**).

**Table 3:** The performance of SRBreak on simulated data sets of different sample sizes.

	TPR			FDR		
**Paired-end read**
Sample size	25.00%	50.00%	75.00%	25.00%	50.00%	75.00%
5	0.75	0.88	1.00	0.00	0.17	0.30
10	0.81	0.89	0.95	0.05	0.11	0.19
20	0.89	0.94	0.97	0.03	0.06	0.10
50	0.94	0.96	0.98	0.01	0.02	0.05
100	0.97	0.98	0.98	0.00	0.01	0.01
**Single-end read**	
Sample size	25.00%	50.00%	75.00%	25.00%	50.00%	75.00%
5	0.80	0.90	1.00	0.00	0.00	0.20
10	0.87	0.94	1.00	0.00	0.05	0.06
20	0.94	0.96	1.00	0.00	0.00	0.03
50	0.95	0.98	0.98	0.00	0.00	0.00
100	0.97	0.97	0.98	0.00	0.00	0.00

*Three percentiles (25, 50, 75%) of TPRs and FDRs were calculated from resampled data sets for each sample size. As an example, with paired-end data, for a sample size of 5, the 25, 50, and 75th percentiles of TPRs from 250 resamplings (i.e., randomly taking five samples to analyze from the 120 simulated samples) were 0.75, 0.90, and 1.00, respectively.*

### Comparison with Other Packages

We compared SRBreak with five other packages: Pindel, DELLY, SoftSearch, MATCHCLIP, and CNVnator.

#### Simulated Data

##### The 1 Mb region (paired-end data only)

Firstly, we compared all packages for all 120 simulated paired-end samples (**Table 4**). TPR and FDR for SRBreak were 0.98 and 0 respectively. Of the other packages, Pindel had the best performance, with 0.88 and 0 for TPR and FDR respectively. The TPR of DELLY was also high (0.78) and its FDR was 0.00. Similarly, MATCHCLIP’s FDR was also zero, but its TPR (0.69) was lower than those of Pindel and DELLY. SoftSearch and CNVnator had the lowest performance, with 0.32, 0.12 and 0.61, 0.47 for TPRs and FDRs respectively.

**Table 4:** Results of different pipelines on 120 samples of simulation data on 1 Mb region.

	All 120 samples		Class I: 1–5x		Class II: 6–10x		Class III: 11–15x	
	TPR	FDR	TPR	FDR	TPR	FDR	TPR	FDR
SRBreak	0.98	0.00	0.77	0.16	1.00	0.00	1.00	0.00
Pindel	0.88	0.00	0.31	0.00	0.89	0.15	0.97	0.08
DeLLY	0.78	0.00	0.63	0.00	0.86	0.00	0.86	0.00
Softsearch	0.32	0.61	0.19	0.78	0.59	0.66	0.91	0.58
MATCHCLIP	0.69	0.00	0.29	0.00	0.77	0.00	1.00	0.00
CNVnator	0.12	0.47	0.11	0.50	0.13	0.50	0.13	0.50

*These 120 are also divided into three classes: I, II, III for coverage 1–5, 6–10, and 11–15x respectively.*

We also tested the methods on different read depths. The 120 samples were divided into three classes with 40 samples for each class (I) 1 to 5x, (II) 6 to 10x, and (III) 11 to 15x. As expected, TPRs increased and FDRs decreased from I to III (**Table 4**). For read depth between 11 and 15x, TPRs of five packages SRBreak, Pindel, DELLY, MATCHCLIP, and Softsearch were very high (≥0.86); the first four packages had low FDRs (≤0.08) while Softsearch’s FDR was very high (0.58). For class II, while TPRs of SRBreak, Pindel, and DELLY were still high (≥0.86), TPRs of MATCHCLIP and Softsearch went down to ≤0.77. For class I, only SRBreak and DELLY had relatively high TPRs (0.77 and 0.63 respectively), other packages had TPRs less than 0.5. CNVnator which is solely based on read counts had TPRs ≤ 0.13 and FDRs of approximately 0.5 for all classes.

##### Chromosome 21 simulation

We also tested SRBreak and other packages on five samples of low-coverage simulation data using the entirety of chromosome 21. In addition, run time was also tested. SRBreak had the highest TRP (0.92) followed by Delly (0.86) and Pindel (0.84). Both SoftSearch and MATCHCLIP had TPRs ≤ 0.2, and also had very high FDRs (0.88 and 0.58 respectively). SRBreak had FDR ∼0.1 while zero FDRs were observed for both Pindel and Delly. CNVnator’s TPR and FDR were 0.01 and 0.99 respectively (**Table 5**). Of all these packages, MATCHCLIP had the lowest run time (less than 1 min), followed by Softsearch, SRBreak, CNVnator (all less than 3 min). DELLY and Pindel had longer run times (>3 min and >3 h respectively); this was likely because these methods had to re-align reads to obtain accurate breakpoints.

**Table 5:** Results of different pipelines on five low-coverage simulated samples (1–5x) for whole chromosome 21.

	Whole chromosome 21		
	TPR	FDR	Time
SRBreak	0.92	0.10	1 m 35 s
Pindel	0.84	0.00	>3 h
DELLY	0.86	0.00	3 m 32 s
SoftSearch	0.20	0.88	1 m 27 s
MATCHCLIP	0.20	0.58	0 m 53 s
CNVnator	0.01	0.99	2 m 15 s

#### Real Data

SRBreak, Pindel, DELLY, MATCHCLIP, and SoftSearch were used to detect the SVs initially identified by McCarroll et al. (2008) and The 1000 Genomes Project (2012) at loci whose CNVs have been associated with increased risks of complex diseases, as described in **Supplementary Table S1**. For SRBreak, the “countThreshold” parameter was set to equal 0.1× sampleSize (i.e., 10% of the number of samples) in order for the package to be able to call events occurring with at least 10% prevalence in the population. A window size of 500 bp was used, and all other parameters remained the same as those used in simulated data sets. All tools were used to analyze 415 samples from four populations downloaded from the 1000 Genomes Project. These 415 samples overlapped with 354 samples analyzed by The 1000 Genomes Project (2012) and 270 samples analyzed by McCarroll et al. (2008), as shown in **Supplementary Table S1**. The CR was used to compare the five pipelines with previous results.

##### NEGR1 region

Results from The 1000 Genomes Project (2012) were used for this locus. All pipelines were able to detect the two SVs at this locus in at least some of the samples (**Table 6**). The highest proportion was seen for Pindel (0.93), followed by SRBreak (0.92) and DELLY (0.90). SoftSearch had the lowest proportion (0.10) while MATCHCLIP reported 47% of breakpoints analyzed by The 1000 Genomes Project (2012).

**Table 6:** The performance of the five analysis methods on three loci.

	NEGR1	LCE3	IRGM
SRBreak	0.92 (344/374)	1.00 (271/271)	0.82 (196/240)
Pindel	0.93 (348/374)	0.85 (231/271)	0.78 (187/240)
DELLY	0.90 (337/374)	0.83 (224/271)	0.68 (163/240)
SoftSearch	0.10 (37/374)	0.1 (27/271)	0.19 (46/240)
MATCHCLIP	0.47 (174/374)	0 (0/271)	0.04 (9/240)

*The table describes the concordance rates between pipelines and results analyzed by McCarroll et al. (2008) and The 1000 Genomes Project (2012). For example, for the two structural variants at the NEGR1 locus, SRBreak detected 344 of the 374 deletions detected by The 1000 Genomes Project (**Supplementary Table S1**).*

##### IRGM region

The results from both McCarroll et al. (2008) and The 1000 Genomes Project (2012) were used for the IRGM locus. All pipelines were able to report the two SVs in this region (**Table 6**). Similar to the NEGR1 locus, SRBreak, Pindel and DELLY showed high concordances with previous results reported at this locus. SRBreak had the highest proportion (0.82), followed by Pindel (0.78) and DELLY (0.68). Detection rates of less than 0.2 were seen for both SoftSearch and MATCHCLIP.

##### LCE3 region

Results from The 1000 Genomes Project (2012) were used for this locus. The SV chr1:152,760,345-152,770,828 (see Methods and Data; outside the LCE3 locus of interest but within the surrounding megabase of DNA sequence) was not reported by any of the tools, therefore it was removed from the calculations. SRBreak was able to report all deletion results from The 1000 Genomes Project (2012) while both Pindel and DELLY reported over 83% of these deletion results. SoftSearch had low concordance for this locus (0.19) while MATCHCLIP did not report any deletions around the radius of 10 bp from breakpoints of the 1000 Genomes (**Table 6**).

##### Performance of SRBreak with mappability information on real data

We tested SRBreak with different thresholds of mapping qualities and corrected read counts with a mappability file. For two loci, LCE3 and IRGM, the concordances at QUAL = 1 and QUAL = 10 were similar to concordances observed when running SRBreak without filtering low-quality reads (**Table 6**; **Supplementary Table S6**). However, for the NEGR1 locus, these concordances were lower than those with no filtering performed (**Table 6**; **Supplementary Table S6**).

## Discussion

Here, an integrated framework to identify breakpoints relating to duplication and deletion events at simple loci has been developed. The pipeline (SRBreak) is based on the combination of read-depth and split-read approaches, with paired-end information able to be used if available. One of the advantages of the pipeline is that it can pinpoint breakpoints for very low-coverage samples if there is sufficient split-read information from samples in the CN group. For example, if a CN group of multiple samples only has reliable split-read information from one sample, then this group’s CN breakpoints can still be inferred using the split-read information from this one sample. The work presented here also showed very high performance for SRBreak on single-end reads, which gives it an advantage over the other four breakpoint detection methods that were investigated, as they only utilize paired-end data. These results show that SRBreak can be successfully used in studies in which both single and paired reads are produced from multiple low-coverage samples or a few high-coverage samples. This approach provides a substantial improvement over our previous methodology, the read-depth based CNVrd2 (Nguyen et al., 2014), through the inclusion of split-read information and the borrowing of read-depth and split-read information across samples to obtain precise breakpoints of a CNVR. As demonstrated here via the CNVnator results, methods which utilize multiple approaches for CNV detection tend to outperform the more simple read-depth based approaches, particularly in regions involving complex CN patterns.

SRBreak was applied to simulated and real data with sample sizes ≥ 120, and compared to five other packages: Pindel (Ye et al., 2009), DELLY (Rausch et al., 2012), MATCHCLIP (Wu et al., 2013) and SoftSearch (Hart et al., 2013), which also use a split-read signature to infer breakpoints; and a read-depth package, CNVnator (Abyzov et al., 2011). SRBreak’s TPRs were comparable with other methods, and its FDR was lower than both MATCHCLIP and SoftSearch. We also tested SRBreak and other packages for whole chromosome 21 on five simulated low-coverage samples (1–5x). Similar results were also observed for the six packages (**Table 5**). Generally SRBreak performed as well as Pindel and DELLY, and all three approaches were clearly superior to Softsearch and MATCHCLIP at the tested loci. SRBreak should be a useful tool to apply to single or small numbers of relatively simple loci and in this context is a good alternative to the multi-tool (*n* = 19) approach used by The 1000 Genomes Project (2012).

SRBreak uses read-depth information as the primary strategy to detect duplication/deletion events. Therefore, it still suffers biases inherent to read-depth-based pipelines, such as mappability or GC-content. As can be seen in **Tables 4** and **5**, even though SRBreak has very high TPRs on simulated data, its FDR was not zero as were the FDR of two paired-end tools DELLY and Pindel. Another weakness of read-depth methods is that their results are affected by window size. Predicted breakpoints can be a window-size distant from real breakpoints. As can be seen in **Tables 4** and **5**, CNVnator, which is purely based on read depth, compares poorly with other methods in predicting breakpoints. In our work, large windows were used in order to obtain reliable results for single-end and low-coverage samples. This can result in missing small CNVRs. This situation can even happen for large SVs if window sizes are higher or equal to the sizes of the SVs. For example, in simulated data (**Table 2**), when the window size is increased to 1000 bp, SRBreak was not able to call the 1000-bp SV for some low-coverage samples. Therefore, in high-coverage work, an adjustment for window sizes should be used to obtain small CNVRs. As discussed by Alkan et al. (2011), read-depth approaches usually result in low-resolution breakpoints. However, integrating split-read information as done in SRBreak can help to alleviate this issue.

From this work, we have demonstrated that the use of multiple samples (SRBreak, DELLY, Pindel) can help in obtaining more reliable structural-variant results, and the strategy of using group information to impute breakpoints of other samples may lead to better results (SRBreak). One of the key features of SRBreak is the use of a threshold to identify CNV regions (countThreshold in the SRBreak package), although setting this threshold is not straightforward – if this value is set too high then some rare regions will be missed, and if it is too low then SRBreak can report non-exact breakpoints because of insufficient split-read information or read-depth information noise. Based on the fact that SRBreak was designed for common CN events, we suggest that this value should be set high (as we did in real data sets analyzed in this study) to obtain more exact breakpoints.

A kernel-based approach was used by SRBreak to obtain precise breakpoints (**Step 2** and **Step 3** in see Methods and Data). In this approach, *σ* values play a central role. For the read-depth-based approach, one-third of the window size was used in this study to capture noisy signals which occur over a distance equal to the width of a single window. For the split-read based approach, as can be seen in **Supplementary Table S3**, split-read positions can occur at or near real breakpoints. It is challenging to understand the distribution of split-read information because it can be influenced by other small variants such as SNPs or INDELs in neighboring regions (e.g., small indels in some samples at the boundaries of a large SV can result in split-read positions around these indels). Moreover, micro-homologies at breakpoints (Conrad et al., 2010; Ottaviani et al., 2014; Abyzov et al., 2015) or CNVRs on segmental duplications (Arlt et al., 2012) can make the dispersal of split-read positions wider. We used σ^S^ = 0.5 to capture only split-read information around the position being calculated, and to avoid capturing other noisy signal far away from the position. However, this small σ^S^ might result in losing split-read information in more complex regions.

Read-depth and split-read information were combined to infer breakpoints at specific CNVRs. As discussed by previous studies (Teo et al., 2012; Sims et al., 2014), read counts can be influenced by GC-content, mappability, and other factors. These issues can then lead to non-reliable outputs from the segmentation and clustering processes. As a result, non-exact breakpoints can be reported, especially in complex regions. Determining how best to remove these biases remains a challenge. In our work, all available reads were utilized by SRBreak to maximize the amount of information being used, and high concordance with previous studies was observed. However, SRBreak also has an option to correct mappability bias if low-quality reads are removed.

In summary, we have proposed a new CNV detection methodology, and compared our work with five other packages using simulated data for a 1 Mb region and for the whole of chromosome 21, along with real data from three known CNV loci. Our package showed higher performance than other packages, especially compared to a purely read-depth package. Even though we simulated different combinations of deletions and duplications within the 1 Mb region for 120 samples, and deletions for five low-coverage samples, the results presented here only pertain to these data sets. SRBreak (and the other methods tested) may show different performance on other data sets; however, we believe that integrating split-read information into a read-depth approach can result in more reliable analysis of CNVRs.

The package is available online at https://github.com/hoangtn/SRBreak. Even though the package can be used to analyze for a single sample, we suggest that people should use multiple samples (at least 5) as in our current study.

## Author Contributions

Designed the pipeline used in analysis: HN, MB. Conceived and designed the experiments: HN, TM, MB. Performed the experiments: HN. Analyzed the data: NH, JB, TM, MB. Contributed reagents/materials/analysis tools: HN, JB. Wrote the paper: HN, TM, MB.

## Conflict of Interest Statement

The authors declare that the research was conducted in the absence of any commercial or financial relationships that could be construed as a potential conflict of interest.

## Footnotes

1 https://github.com/nh13/dwgsim

2 ftp://ftp.1000genomes.ebi.ac.uk/vol1/ftp/release/20130502/

## Supplementary Material

FIGURE S1 **Different events in the simulated data sets.** For example, the top left picture shows a small deletion event (1000 bp) while the bottom left picture shows a large duplication event (50,000 bp). See also **Supplementary Table S1**.

FIGURE S2 **An example describing the use of split-read information to obtain more reliable breakpoints for four samples in the same group at the NEGR1 locus.** The x axis depicts genome coordinates while the y axis shows the occurrences of split-read positions. Red vertical lines are breakpoint positions reported by The 1000 Genomes Project (2012).

TABLE S1 **Breakpoints of deletion regions (allele frequencies > 0.0025, lengths > 500) reported by McCarroll et al. (2008) (^∗∗^) and The 1000 Genomes Project (2012) (^∗^) on 1 Mb regions around the three loci IRGM, NEGR1, LCE3.** There were 1092 samples analyzed by The 1000 Genomes Project (2012), and 270 analyzed by McCarroll et al. (2008). In this study, the 415 most recent samples from four populations: CEU, YRI, CHB, and JPT were downloaded and analyzed using five different CN detection pipelines. The sample sizes presented in the table are the overlapping sample sizes between the 415 and the 1092 from The 1000 Genomes Project (2012) or between the 415 and the 270 of McCarroll et al. (2008). The structural variant chr1:152,760,345-152,770,828 at the LCE3 locus was removed from this study because it was not reported by any of the pipelines used, or in the recent analysis of the same 1092 samples by Abyzov et al. (2015). The results of McCarroll et al. (2008) were only used for one event (chr5:150203163-150223264) at the IRGM locus.

TABLE S2 **(A)** Notation used in the SRBreak analysis pipeline. **(B)** The performance of different models for different window sizes (W). Names of the models are from the mclust package. If the clustering process could not be executed for the model then the corresponding cell is empty. **(C)** Results for simulated data using different σ and a simple approach to obtain breakpoints for the read-depth based step in SRBreak on 1 Mb region (120 samples). σ values range from 1/10 to 2 times of the window size (W/10 to W^∗^2).

TABLE S3 **Illustrative CIGAR strings in BED files around two breakpoints, 101,545,220 and 101,630,000, in the simulated data.** For example, the CIGAR string 76M24S in the first row describes a mapped read whose length is 100 bp: 24 bp was not mapped (24S) and 76 bp was mapped (76 M). The start position of the mapped part was 101,545,143. Split positions can be seen around the two breakpoints, therefore, a kernel-based approach can be used to obtain all split-read information. For deleted events, only split positions of the Left Group are used to infer left breakpoints and only split positions of the Right Group are used to infer right breakpoints. For duplicated events, all split positions of the Left and Right Groups are used for both left and right breakpoints.

TABLE S4 **The distances between real breakpoints and predicted breakpoints on 120 simulated samples.** For SRBreak, this information is for both single-end and paired-end samples. For other pipelines, only paired-end information was described. The results are events overlapping with simulated structural variants.

TABLE S5 **The results of the 1000 bp event called by SRBreak for 15 duplicated samples.** The best results were seen for window sizes of 500 bp: the package called exactly 13/15 duplication samples [failed to called samples of low (1–2x) coverage].

TABLE S6 **Concordant results between SRBreak and McCarroll et al. (2008) and The 1000 Genomes Project (2012) for three loci NEGR1, LCE3, and IRGM with different mapping qualities (QUAL).** These results were obtained by running SRBreak with a mappability file and removing reads having QUALs lower than 1, 10, or 20 as described in columns.

## References

[B1] Abyzov A. Li S. Kim D. R. Mohiyuddin M. Stutz A. M. Parrish N. F. (2015). Analysis of deletion breakpoints from 1,092 humans reveals details of mutation mechanisms. *Nat. Commun.* 6 7256 10.1038/ncomms8256 PMC4451611 26028266

[B2] Abyzov A. Urban A. E. Snyder M. Gerstein M. (2011). CNVnator: an approach to discover, genotype, and characterize typical and atypical CNVs from family and population genome sequencing. *Genome Res.* 21 974–984. 10.1101/gr.114876.110 21324876 PMC3106330

[B3] Aklillu E. Odenthal-Hesse L. Bowdrey J. Habtewold A. Ngaimisi E. Yimer G. (2013). CCL3L1 copy number, HIV load, and immune reconstitution in sub-Saharan Africans. *BMC Infect. Dis.* 13: 536 10.1186/1471-2334-13-536 PMC3829100 24219137

[B4] Alkan C. Coe B. P. Eichler E. E. (2011). Genome structural variation discovery and genotyping. *Nat. Rev. Genet.* 12 363–376. 10.1038/nrg2958 21358748 PMC4108431

[B5] Arlt M. F. Wilson T. E. Glover T. W. (2012). Replication stress and mechanisms of CNV formation. *Curr. Opin. Genet. Dev.* 22 204–210. 10.1016/j.gde.2012.01.009 22365495 PMC3371136

[B6] Bailey J. A. Yavor A. M. Massa H. F. Trask B. J. Eichler E. E. (2001). Segmental duplications: organization and impact within the current human genome project assembly. *Genome Res.* 11 1005–1017. 10.1101/gr.187101 11381028 PMC311093

[B7] Bentley R. W. Pearson J. Gearry R. B. Barclay M. L. McKinney C. Merriman T. R. (2009). Association of higher DEFB4 genomic copy number with Crohn’s disease. *Am. J. Gastroenterol.* 105 354–359. 10.1038/ajg.2009.582 19809410

[B8] Carpenter D. Walker S. Prescott N. Schalkwijk J. Armour J. A. L. (2011). Accuracy and differential bias in copy number measurement of CCL3L1 in association studies with three auto-immune disorders. *BMC Genomics* 12:418 10.1186/1471-2164-12-418 PMC3166952 21851606

[B9] Chen K. Wallis J. W. McLellan M. D. Larson D. E. Kalicki J. M. Pohl C. S. (2009). BreakDancer: an algorithm for high-resolution mapping of genomic structural variation. *Nat. Methods* 6 677–681. 10.1038/nmeth.1363 19668202 PMC3661775

[B10] Chung B. H.-Y. Tao V. Q. Tso W. W.-Y. (2014). Copy number variation and autism: new insights and clinical implications. *J. Formos. Med. Assoc.* 113 400–408. 10.1016/j.jfma.2013.01.005 24961180

[B11] Conrad D. F. Pinto D. Redon R. Feuk L. Gokcumen O. Zhang Y. (2010). Origins and functional impact of copy number variation in the human genome. *Nature* 464 704–712. 10.1038/nature08516 19812545 PMC3330748

[B12] Danecek P. Auton A. Abecasis G. Albers C. A. Banks E. DePristo M. A. (2011). The variant call format and VCFtools. *Bioinformatics* 27 2156–2158. 10.1093/bioinformatics/btr330 21653522 PMC3137218

[B13] de Cid R. Riveira-Munoz E. Zeeuwen P. L. Robarge J. Liao W. Dannhauser E. N. (2009). Deletion of the late cornified envelope LCE3B and LCE3C genes as a susceptibility factor for psoriasis. *Nat. Genet.* 41 211–215. 10.1038/ng.313 19169253 PMC3128734

[B14] Falchi M. Moustafa J. S. E.-S. Takousis P. Pesce F. Bonnefond A. Andersson-Assarsson J. C. (2014). Low copy number of the salivary amylase gene predisposes to obesity. *Nat. Genet.* 46 492–497. 10.1038/ng.2939 24686848 PMC6485469

[B15] Fraley C. Raftery A. (2009). mclust: Model-based clustering/normal mixture modeling. *R package version 3(1)*.

[B16] Fraley C. Raftery A. E. (1998). How many clusters? Which clustering method? Answers via model-based cluster analysis. *Comput. J.* 41 578–588.

[B17] Gonzalez E. Kulkarni H. Bolivar H. Mangano A. Sanchez R. Catano G. (2005). The influence of CCL3L1 gene-containing segmental duplications on HIV-1/AIDS susceptibility. *Science* 307 1434–1440. 10.1126/science.1101160 15637236

[B18] Green E. Rees E. Walters J. Smith K. Forty L. Grozeva D. (2016). Copy number variation in bipolar disorder. *Mol. psychiatry* 21 89–93. 10.1038/mp.2014.174 25560756 PMC5038134

[B19] Hardwick R. J. Machado L. R. Zuccherato L. W. Antolinos S. Xue Y. Shawa N. (2011). A worldwide analysis of beta-defensin copy number variation suggests recent selection of a high-expressing DEFB103 gene copy in East Asia. *Hum. Mutat.* 32 743–750. 10.1002/humu.21491 21387465 PMC3263423

[B20] Hardwick R. J. Ménard A. Sironi M. Milet J. Garcia A. Sese C. (2014). Haptoglobin (HP) and Haptoglobin-related protein (HPR) copy number variation, natural selection, and trypanosomiasis. *Hum. Genet.* 133 69–83. 10.1007/s00439-013-1352-x 24005574 PMC3898332

[B21] Hart S. N. Sarangi V. Moore R. Baheti S. Bhavsar J. D. Couch F. J. (2013). SoftSearch: integration of multiple sequence features to identify breakpoints of structural variations. *PLoS ONE* 8:e83356 10.1371/journal.pone.0083356 PMC3865185 24358278

[B22] He W. Kulkarni H. Castiblanco J. Shimizu C. Aluyen U. Maldonado R. (2009). Reply to: “Experimental aspects of copy number variant assays at CCL3L1”. *Nat. Med.* 15 1117–1120. 10.1038/nm1009-1117 19812563

[B23] Hooli B. Kovacs-Vajna Z. M. Mullin K. Blumenthal M. Mattheisen M. Zhang C. (2014). Rare autosomal copy number variations in early-onset familial Alzheimer’s disease. *Mol. Psychiatry* 19 676–681. 10.1038/mp.2013.77 23752245

[B24] Jiang Y. Wang Y. Brudno M. (2012). PRISM: pair-read informed split-read mapping for base-pair level detection of insertion, deletion and structural variants. *Bioinformatics* 28 2576–2583. 10.1093/bioinformatics/bts484 22851530

[B25] Karolchik D. Barber G. P. Casper J. Clawson H. Cline M. S. Diekhans M. (2014). The UCSC genome browser database: 2014 update. *Nucleic Acids Res.* 42(Database issue) D764–D770. 10.1093/nar/gkt1168 24270787 PMC3964947

[B26] Kidd J. M. Cooper G. M. Donahue W. F. Hayden H. S. Sampas N. Graves T. (2008). Mapping and sequencing of structural variation from eight human genomes. *Nature* 453 56–64. 10.1038/nature06862 18451855 PMC2424287

[B27] Koboldt D. C. Zhang Q. Larson D. E. Shen D. McLellan M. D. Lin L. (2012). VarScan 2: somatic mutation and copy number alteration discovery in cancer by exome sequencing. *Genome Res.* 22 568–576. 10.1101/gr.129684.111 22300766 PMC3290792

[B28] Korbel J. O. Urban A. E. Affourtit J. P. Godwin B. Grubert F. Simons J. F. (2007). Paired-end mapping reveals extensive structural variation in the human genome. *Science* 318 420–426. 10.1126/science.1149504 17901297 PMC2674581

[B29] Layer R. M. Chiang C. Quinlan A. R. Hall I. M. (2014). LUMPY: a probabilistic framework for structural variant discovery. *Genome Biol.* 15 R84 10.1186/gb-2014-15-6-r84 PMC4197822 24970577

[B30] Li H. (2011). Tabix: fast retrieval of sequence features from generic TAB-delimited files. *Bioinformatics* 27 718–719. 10.1093/bioinformatics/btq671 21208982 PMC3042176

[B31] Li H. (2013). *Aligning Sequence Reads, Clone Sequences and Assembly Contigs with BWA-MEM*. Available at: http://arxiv.org/abs/1303.3997v2

[B32] Li H. Handsaker B. Wysoker A. Fennell T. Ruan J. Homer N. (2009). The sequence alignment/map format and SAMtools. *Bioinformatics* 25 2078–2079. 10.1093/bioinformatics/btp352 19505943 PMC2723002

[B33] Lindsay S. J. Khajavi M. Lupski J. R. Hurles M. E. (2006). A chromosomal rearrangement hotspot can be identified from population genetic variation and is coincident with a hotspot for allelic recombination. *Am. J. Hum. Genet.* 79 890–902. 10.1086/508709 17033965 PMC1698570

[B34] McCarroll S. A. Huett A. Kuballa P. Chilewski S. D. Landry A. Goyette P. (2008). Deletion polymorphism upstream of IRGM associated with altered IRGM expression and Crohn’s disease. *Nat. Genet.* 40 1107–1112. 10.1038/ng.215 19165925 PMC2731799

[B35] McKinney C. Fanciulli M. Merriman M. E. Phipps-Green A. Alizadeh B. Z. Koeleman B. P. (2010). Association of variation in Fcgamma receptor 3B gene copy number with rheumatoid arthritis in Caucasian samples. *Ann. Rheum. Dis.* 69 1711–1716. 10.1136/ard.2009.123588 20472591 PMC3670580

[B36] McKinney C. Merriman T. R. (2012). Meta-analysis confirms a role for deletion in FCGR3B in autoimmune phenotypes. *Hum. Mol. Genet.* 21 2370–2376. 10.1093/hmg/dds039 22337955

[B37] Mueller M. Barros P. Witherden A. S. Roberts A. L. Zhang Z. Schaschl H. (2013). Genomic pathology of SLE-associated copy-number variation at the FCGR2C/FCGR3B/FCGR2B locus. *Am. J. Hum. Genet.* 92 28–40. 10.1016/j.ajhg.2012.11.013 23261299 PMC3542466

[B38] Nguyen H. T. Merriman T. R. Black M. A. (2013). CNVrd, a read-depth algorithm for assigning copy-number at the FCGR locus: population-specific tagging of copy number variation at FCGR3B. *PLoS ONE* 8:e63219 10.1371/journal.pone.0063219 PMC3640002 23646200

[B39] Nguyen H. T. Merriman T. R. Black M. A. (2014). The CNVrd2 package: measurement of copy number at complex loci using high-throughput sequencing data. *Front. Genet.* 5:248 10.3389/fgene.2014.00248 PMC4117933 25136349

[B40] Nordang G. B. Carpenter D. Viken M. K. Kvien T. K. Armour J. A. Lie B. A. (2012). Association analysis of the CCL3L1 copy number locus by paralogue ratio test in Norwegian rheumatoid arthritis patients and healthy controls. *Genes Immun.* 13 579–582. 10.1038/gene.2012.30 22785612

[B41] Olson H. Shen Y. Avallone J. Sheidley B. R. Pinsky R. Bergin A. M. (2014). Copy number variation plays an important role in clinical epilepsy. *Ann. Neurol.* 75 943–958. 10.1002/ana.24178 24811917 PMC4487364

[B42] Ottaviani D. LeCain M. Sheer D. (2014). The role of microhomology in genomic structural variation. *Trends Genet.* 30 85–94. 10.1016/j.tig.2014.01.001 24503142

[B43] Perry G. H. Dominy N. J. Claw K. G. Lee A. S. Fiegler H. Redon R. (2007). Diet and the evolution of human amylase gene copy number variation. *Nat. Genet.* 39 1256–1260. 10.1038/ng2123 17828263 PMC2377015

[B44] Rausch T. Zichner T. Schlattl A. Stutz A. M. Benes V. Korbel J. O. (2012). DELLY: structural variant discovery by integrated paired-end and split-read analysis. *Bioinformatics* 28 i333–i339. 10.1093/bioinformatics/bts378 22962449 PMC3436805

[B45] Redon R. Ishikawa S. Fitch K. R. Feuk L. Perry G. H. Andrews T. D. (2006). Global variation in copy number in the human genome. *Nature* 444 444–454. 10.1038/nature05329 17122850 PMC2669898

[B46] Schwarz G. (1978). Estimating the dimension of a model. *Ann. Stat.* 6 461–464. 10.1214/aos/1176344136

[B47] Shrestha S. Nyaku M. Edberg J. C. (2010). Variations in CCL3L gene cluster sequence and non-specific gene copy numbers. *BMC Res. Notes* 3:74 10.1186/1756-0500-3-74 PMC2851716 20233400

[B48] Sims D. Sudbery I. Ilott N. E. Heger A. Ponting C. P. (2014). Sequencing depth and coverage: key considerations in genomic analyses. *Nat. Rev. Genet.* 15 121–132. 10.1038/nrg3642 24434847

[B49] Teo S. M. Pawitan Y. Ku C. S. Chia K. S. Salim A. (2012). Statistical challenges associated with detecting copy number variations with next-generation sequencing. *Bioinformatics* 28 2711–2718. 10.1093/bioinformatics/bts535 22942022

[B50] The 1000 Genomes Project (2010). A map of human genome variation from population-scale sequencing. *Nature* 467 1061–1073. 10.1038/nature09534 20981092 PMC3042601

[B51] The 1000 Genomes Project (2012). An integrated map of genetic variation from 1,092 human genomes. *Nature* 491 56–65. 10.1038/nature11632 23128226 PMC3498066

[B52] Tuzun E. Sharp A. J. Bailey J. A. Kaul R. Morrison V. A. Pertz L. M. (2005). Fine-scale structural variation of the human genome. *Nat. Genet.* 37 727–732. 10.1038/ng1562 15895083

[B53] Veal C. D. Reekie K. E. Lorentzen J. C. Gregersen P. K. Padyukov L. Brookes A. J. (2014). A 129-kb deletion on chromosome 12 confers substantial protection against rheumatoid arthritis, implicating the gene SLC2A3. *Hum. Mutat.* 35 248–256. 10.1002/humu.22471 24178905 PMC3995011

[B54] Wang J. Mullighan C. G. Easton J. Roberts S. Heatley S. L. Ma J. (2011). CREST maps somatic structural variation in cancer genomes with base-pair resolution. *Nat. Methods* 8 652–654. 10.1038/nmeth.1628 21666668 PMC3527068

[B55] Wang Z. Hormozdiari F. Yang W.-Y. Halperin E. Eskin E. (2013). CNVeM: copy number variation detection using uncertainty of read mapping. *J. Comput. Biol.* 20 224–236. 10.1089/cmb.2012.0258 23421794 PMC3590897

[B56] Willer C. J. Speliotes E. K. Loos R. J. Li S. Lindgren C. M. Heid I. M. (2009). Six new loci associated with body mass index highlight a neuronal influence on body weight regulation. *Nat. Genet.* 41 25–34. 10.1038/ng.287 19079261 PMC2695662

[B57] Wong K. Keane T. M. Stalker J. Adams D. J. (2010). Enhanced structural variant and breakpoint detection using SVMerge by integration of multiple detection methods and local assembly. *Genome Biol.* 11 R128 10.1186/gb-2010-11-12-r128 PMC3046488 21194472

[B58] Wu Y. Tian L. Pirastu M. Stambolian D. Li H. (2013). MATCHCLIP: locate precise breakpoints for copy number variation using CIGAR string by matching soft clipped reads. *Front. Genet.* 4:157 10.3389/fgene.2013.00157 PMC3744852 23967014

[B59] Ye K. Schulz M. H. Long Q. Apweiler R. Ning Z. (2009). Pindel: a pattern growth approach to detect break points of large deletions and medium sized insertions from paired-end short reads. *Bioinformatics* 25 2865–2871. 10.1093/bioinformatics/btp394 19561018 PMC2781750

[B60] Yoon S. Xuan Z. Makarov V. Ye K. Sebat J. (2009). Sensitive and accurate detection of copy number variants using read depth of coverage. *Genome Res.* 19 1586–1592. 10.1101/gr.092981.109 19657104 PMC2752127

[B61] Zeitouni B. Boeva V. Janoueix-Lerosey I. Loeillet S. Legoix-Né P. Nicolas A. (2010). SVDetect: a tool to identify genomic structural variations from paired-end and mate-pair sequencing data. *Bioinformatics* 26 1895–1896. 10.1093/bioinformatics/btq293 20639544 PMC2905550

[B62] Zhao M. Wang Q. Jia P. Zhao Z. (2013). Computational tools for copy number variation (CNV) detection using next-generation sequencing data: features and perspectives. *BMC Bioinform.* 14(Suppl. 11):S1 10.1186/1471-2105-14-S11-S1 PMC3846878 24564169

