# Supplementary figures and images for: SRBreak: A Read-Depth and Split-Read Framework to Identify Breakpoints of Different Events Inside Simple Copy-Number Variable Regions
Source: Front Genet. 2016 Sep 15;7:160. doi: 10.3389/fgene.2016.00160 (PMC5023681; doi:10.3389/fgene.2016.00160)

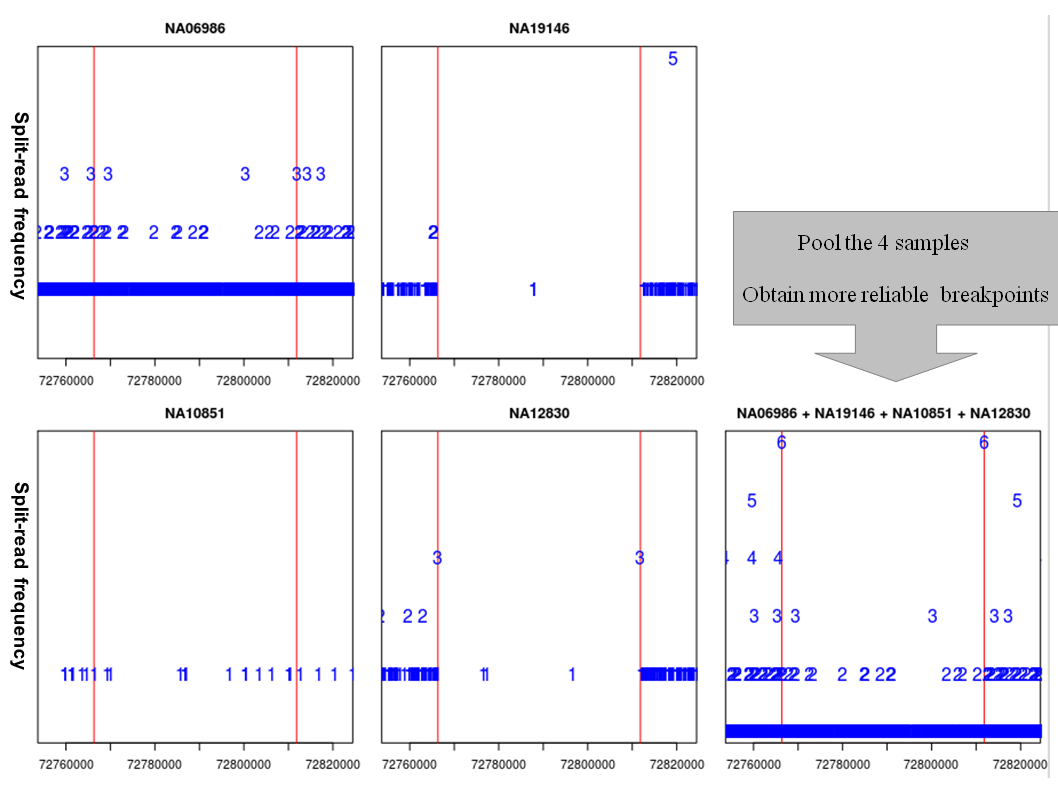

Supplement: FIGURE S2 — An example describing the use of split-read information to obtain more reliable breakpoints for four samples in the same group at the NEGR1 locus. The x axis depicts genome coordinates while the y axis shows the occurrences of split-read positions. Red vertical lines are breakpoint positions reported by The 1000 Genomes Project (2012). [file Image_2.TIF]
