# Supplementary material for: SRBreak: A Read-Depth and Split-Read Framework to Identify Breakpoints of Different Events Inside Simple Copy-Number Variable Regions
Source: Front Genet. 2016 Sep 15;7:160. doi: 10.3389/fgene.2016.00160 (PMC5023681; doi:10.3389/fgene.2016.00160)
Supplement: TABLE S1 — Breakpoints of deletion regions (allele frequencies > 0.0025, lengths > 500) reported by McCarroll et al. (2008) (∗∗) and The 1000 Genomes Project (2012) (∗) on 1 Mb regions around the three loci IRGM, NEGR1, LCE3. There were 1092 samples analyzed by The 1000 Genomes Project (2012), and 270 analyzed by McCarroll et al. (2008). In this study, the 415 most recent samples from four populations: CEU, YRI, CHB, and JPT were downloaded and analyzed using five different CN detection pipelines. The sample sizes presented in the table are the overlapping sample sizes between the 415 and the 1092 from The 1000 Genomes Project (2012) or between the 415 and the 270 of McCarroll et al. (2008). The structural variant chr1:152,760,345-152,770,828 at the LCE3 locus was removed from this study because it was not reported by any of the pipelines used, or in the recent analysis of the same 1092 samples by Abyzov et al. (2015). The results of McCarroll et al. (2008) were only used for one event (chr5:150203163-150223264) at the IRGM locus. [file Table_1.DOCX]

**S1 Table**

| Locus | Start | End | Length | Sample Size | Number of Deletion | Used in this study | Allele frequency | Previous results |
| --- | --- | --- | --- | --- | --- | --- | --- | --- |
| IRGM | 150177661 | 150181599 | 3938 | 354 | 143 | Yes | 0.19 | (*) |
|  | 150203163 | 150223264 | 20101 | 180 | 83 | Yes | 0.3 | (**) |
|  |  |  |  |  |  |  |  |  |
| NEGR1 | 72755692 | 72764074 | 8382 | 354 | 49 | Yes | 0.1 | (*) |
|  | 72766323 | 72811839 | 45516 | 354 | 325 | Yes | 0.7 | (*) |
|  |  |  |  |  |  |  |  |  |
| LCE3 | 152555542 | 152587742 | 32200 | 354 | 271 | Yes | 0.58 | (*) |
|  | 152760345 | 152770828 | 10483 | - | - | - | 0.22 | (*) |
