# Supplementary material for: SRBreak: A Read-Depth and Split-Read Framework to Identify Breakpoints of Different Events Inside Simple Copy-Number Variable Regions
Source: Front Genet. 2016 Sep 15;7:160. doi: 10.3389/fgene.2016.00160 (PMC5023681; doi:10.3389/fgene.2016.00160)
Supplement: TABLE S2 — (A) Notation used in the SRBreak analysis pipeline. (B) The performance of different models for different window sizes (W). Names of the models are from the mclust package. If the clustering process could not be executed for the model then the corresponding cell is empty. (C) Results for simulated data using different σ and a simple approach to obtain breakpoints for the read-depth based step in SRBreak on 1 Mb region (120 samples). σ values range from 1/10 to 2 times of the window size (W/10 to W∗2). [file Table_2.DOCX]

**S2 Table**

**Table S2A**

| Notation | Meaning |
| --- | --- |
| *Window* | Window size |
| *M_g_* | Number of samples in a group |
| *scoreP**_j_^R^* | Read-depth based score at a *jth* position |
| *w_k_^R^* | Read-depth based weight at a *kth* position |
| *σ**^R^* | Standard deviation of a read-depth based kernel |
| *μ_k_^R^* | Mean of a read-depth based kernel at a *kth* position |
| *MedLeft/MedRight* | Read-depth based left/right breakpoint of a group |
| *scoreP_j_^S^* | Split-read based score at a *jth* position |
| *w_k_^S^* | Split-read based weight at a *kth* position |
| *σ^S^* | Standard deviation of a split-read based kernel |
| *µ_k_^S^* | Mean of a split-read based kernel at a *kth* position |
| *ε_Open_* | Maximum distance from *MedLeft/MedRight*to find split-read based breakpoints |
| *N_r_* | Number of the resampling process for a group to identify boundaries for the group using read-depth information. |

Table S2B The performance of different models for different windows (W) with a threshold > 0.25 for duplications and < -0.25 for deletions. Names of the models are from the mclust package. If the clustering process could not be executed for the model then the corresponding cell is empty.

|  |  | W = 50 |  | W = 100 |  | W = 250 |  | W = 500 |  | W = 1000 |  |
| --- | --- | --- | --- | --- | --- | --- | --- | --- | --- | --- | --- |
|  |  | TPR | FDR | TPR | FDR | TPR | FDR | TPR | FDR | TPR | FDR |
| Paired end | Model Name | |  |  |  |  |  |  |  |  |  |
|  | EII | 0.80 | 0.11 | 0.94 | 0.01 | 0.96 | 0.01 | 0.98 | 0.00 | 0.82 | 0.05 |
|  | VII | 0.75 | 0.11 | 0.99 | 0.13 | 0.83 | 0.05 | 0.87 | 0.06 | 0.81 | 0.05 |
|  | EEI | 0.80 | 0.10 | 0.94 | 0.01 | 0.96 | 0.01 | 0.98 | 0.00 | 0.80 | 0.07 |
|  | VEI | 0.58 | 0.25 | 0.99 | 0.13 | 0.29 | 0.60 | 0.87 | 0.06 | 0.81 | 0.05 |
|  | EVI | 0.80 | 0.10 | 0.21 | 0.75 | 0.29 | 0.60 | 0.93 | 0.05 | 0.82 | 0.04 |
|  | VVI | 0.44 | 0.39 | 0.24 | 0.62 | 0.29 | 0.60 | 0.88 | 0.06 | 0.79 | 0.08 |
|  | EVE |  |  |  |  | 0.29 | 0.60 | 0.29 | 0.67 | 0.29 | 0.60 |
|  | VVE |  |  |  |  | 0.29 | 0.60 | 0.29 | 0.67 | 0.29 | 0.60 |
|  | VEV |  |  |  |  | 0.29 | 0.60 | 0.29 | 0.67 | 0.29 | 0.60 |
|  | VVV |  |  |  |  | 0.29 | 0.60 | 0.29 | 0.67 | 0.29 | 0.60 |
|  |  |  |  |  |  |  |  |  |  |  |  |
| Single end |  |  |  |  |  |  |  |  |  |  |  |
|  | EII | 0.58 | 0.10 | 0.71 | 0.01 | 0.97 | 0.00 | 0.97 | 0.00 | 0.86 | 0.00 |
|  | VII | 0.58 | 0.10 | 0.86 | 0.01 | 0.84 | 0.12 | 0.97 | 0.00 | 0.86 | 0.00 |
|  | EEI | 0.32 | 0.16 | 0.86 | 0.00 | 0.97 | 0.00 | 0.97 | 0.00 | 0.86 | 0.00 |
|  | VEI | 0.20 | 0.24 | 0.85 | 0.00 | 0.85 | 0.11 | 0.97 | 0.00 | 0.86 | 0.00 |
|  | EVI | 0.20 | 0.24 | 0.71 | 0.00 | 0.95 | 0.02 | 1.00 | 0.13 | 0.86 | 0.00 |
|  | VVI | 0.20 | 0.24 | 0.43 | 0.00 | 0.85 | 0.11 | 0.97 | 0.00 | 0.86 | 0.00 |
|  | EVE |  |  |  |  | 0.29 | 0.66 | 0.29 | 0.67 | 0.29 | 0.60 |
|  | VVE |  |  |  |  | 0.29 | 0.66 | 0.29 | 0.67 | 0.29 | 0.60 |
|  | VEV |  |  |  |  | 0.29 | 0.66 | 0.29 | 0.67 | 0.29 | 0.60 |
|  | VVV |  |  |  |  | 0.29 | 0.66 | 0.29 | 0.67 | 0.29 | 0.60 |

Table S2C Results for simulated data using different σ and a simple approach to obtain breakpoints for the read-depth based step in SRBreak on 1Mb region (120 samples). σ values range from 1/10 to 2 times of the window size (W/10 to W*2).

| Paired-end read |  | W = 50 |  | W = 100 |  | W = 250 |  | W = 500 |  | W = 1000 |  |
| --- | --- | --- | --- | --- | --- | --- | --- | --- | --- | --- | --- |
|  |  | TPR | FDR | TPR | FDR | TPR | FDR | TPR | FDR | TPR | FDR |
|  | W/10 | 0.80 | 0.11 | 0.94 | 0.01 | 0.96 | 0.01 | 0.98 | 0.00 | 0.82 | 0.04 |
|  | W/4 | 0.80 | 0.11 | 0.94 | 0.01 | 0.96 | 0.01 | 0.98 | 0.00 | 0.82 | 0.04 |
| ***** | **W/3** | **0.80** | **0.11** | **0.94** | **0.01** | **0.96** | **0.01** | **0.98** | **0.00** | **0.82** | **0.05** |
|  | W/2 | 0.80 | 0.11 | 0.93 | 0.01 | 0.96 | 0.01 | 0.98 | 0.01 | 0.82 | 0.05 |
|  | W*1 | 0.61 | 0.06 | 0.80 | 0.01 | 0.96 | 0.01 | 0.98 | 0.00 | 0.82 | 0.05 |
|  | W*2 | 0.55 | 0.15 | 0.52 | 0.02 | 0.83 | 0.01 | 0.84 | 0.00 | 0.81 | 0.06 |
|  | Simple score | 0.84 | 0.06 | 0.93 | 0.01 | 0.96 | 0.01 | 0.98 | 0.00 | 0.81 | 0.06 |
|  |  |  |  |  |  |  |  |  |  |  |  |
| Single-end read | |  |  |  |  |  |  |  |  |  |  |
|  |  | TPR | FDR | TPR | FDR | TPR | FDR | TPR | FDR | TPR | FDR |
|  | W/10 | 0.58 | 0.10 | 0.70 | 0.00 | 0.97 | 0.00 | 0.97 | 0.00 | 0.86 | 0.00 |
|  | W/4 | 0.58 | 0.10 | 0.70 | 0.00 | 0.97 | 0.00 | 0.97 | 0.00 | 0.86 | 0.00 |
| ***** | **W/3** | **0.58** | **0.10** | **0.85** | **0.00** | **0.97** | **0.00** | **0.97** | **0.00** | **0.86** | **0.00** |
|  | W/2 | 0.58 | 0.10 | 0.86 | 0.00 | 0.97 | 0.00 | 0.97 | 0.00 | 0.86 | 0.00 |
|  | W*1 | 0.64 | 0.00 | 0.85 | 0.00 | 0.97 | 0.00 | 0.97 | 0.00 | 0.86 | 0.00 |
|  | W*2 | 0.27 | 0.00 | 0.51 | 0.00 | 0.83 | 0.00 | 0.97 | 0.00 | 0.86 | 0.00 |
|  | Simple score | 0.58 | 0.10 | 0.71 | 0.00 | 0.97 | 0.00 | 0.97 | 0.00 | 0.86 | 0.00 |
