# Supplementary material for: SRBreak: A Read-Depth and Split-Read Framework to Identify Breakpoints of Different Events Inside Simple Copy-Number Variable Regions
Source: Front Genet. 2016 Sep 15;7:160. doi: 10.3389/fgene.2016.00160 (PMC5023681; doi:10.3389/fgene.2016.00160)
Supplement: TABLE S3 — Illustrative CIGAR strings in BED files around two breakpoints, 101,545,220 and 101,630,000, in the simulated data. For example, the CIGAR string 76M24S in the first row describes a mapped read whose length is 100 bp: 24 bp was not mapped (24S) and 76 bp was mapped (76 M). The start position of the mapped part was 101,545,143. Split positions can be seen around the two breakpoints, therefore, a kernel-based approach can be used to obtain all split-read information. For deleted events, only split positions of the Left Group are used to infer left breakpoints and only split positions of the Right Group are used to infer right breakpoints. For duplicated events, all split positions of the Left and Right Groups are used for both left and right breakpoints. [file Table_3.DOCX]

**Table S3**

| Group | Start | End | CIGAR string |
| --- | --- | --- | --- |
| Left | 101545143 | 101545219 | 76M24S |
| Left | 101545144 | 101545220 | 76M24S |
| Left | 101545149 | 101545221 | 72M28S |
|  |  |  |  |
| Right | 101630002 | 101630085 | 17S83M |
| Right | 101630000 | 101630069 | 31S69M |
| Right | 101630001 | 101630089 | 12S88M |
