# Supplementary material for: SRBreak: A Read-Depth and Split-Read Framework to Identify Breakpoints of Different Events Inside Simple Copy-Number Variable Regions
Source: Front Genet. 2016 Sep 15;7:160. doi: 10.3389/fgene.2016.00160 (PMC5023681; doi:10.3389/fgene.2016.00160)
Supplement: TABLE S4 — The distances between real breakpoints and predicted breakpoints on 120 simulated samples. For SRBreak, this information is for both single-end and paired-end samples. For other pipelines, only paired-end information was described. The results are events overlapping with simulated structural variants. [file Table_4.DOCX]

**S4 Table**

| **Real breakpoint** | | **Predicted breakpoint** | | **Distance** | |
| --- | --- | --- | --- | --- | --- |
| **SRBreak** |  |  |  |  |  |
| Single-end read |  |  |  |  |  |
| rStart | rEnd | pStart | pEnd | pStart – rStart | pEnd – rEnd |
| 101545220 | 101630000 | 101545220 | 101630000 | 0 | 0 |
| 101555000 | 101605000 | 101554999 | 101605000 | -1 | 0 |
| 101556000 | 101576000 | 101556000 | 101576004 | 0 | 4 |
| 101556000 | 101576000 | 101556004 | 101576000 | 4 | 0 |
| 101558000 | 101568000 | 101558000 | 101568000 | 0 | 0 |
| 101560000 | 101565000 | 101560000 | 101564999 | 0 | -1 |
| 101561000 | 101562000 | 101561000 | 101562000 | 0 | 0 |
|  |  |  |  |  |  |
| Paired-end read |  |  |  |  |  |
| 101545220 | 101630000 | 101545220 | 101630000 | 0 | 0 |
| 101555000 | 101605000 | 101554999 | 101605000 | -1 | 0 |
| 101556000 | 101576000 | 101556000 | 101576004 | 0 | 4 |
| 101556000 | 101576000 | 101556004 | 101576000 | 4 | 0 |
| 101558000 | 101568000 | 101558000 | 101568000 | 0 | 0 |
| 101560000 | 101565000 | 101560000 | 101564999 | 0 | -1 |
| 101561000 | 101562000 | 101561000 | 101562000 | 0 | 0 |
|  |  |  |  |  |  |
| **DELLY** |  |  |  |  |  |
| 101545220 | 101630000 | 101545220 | 101630000 | 0 | 0 |
| 101555000 | 101605000 | 101554999 | 101604999 | -1 | -1 |
| 101556000 | 101576000 | 101556000 | 101576000 | 0 | 0 |
| 101556000 | 101576000 | 101556000 | 101576000 | 0 | 0 |
| 101558000 | 101568000 | 101558000 | 101568000 | 0 | 0 |
| 101560000 | 101565000 | 101559999 | 101564999 | -1 | -1 |
| 101561000 | 101562000 | 101560748 | 101562000 | -252 | 0 |
|  |  |  |  |  |  |
| **PINDEL** |  |  |  |  |  |
| 101545220 | 101630000 | 101545220 | 101630000 | 0 | 0 |
| 101555000 | 101605000 | 101554999 | 101604999 | -1 | -1 |
| 101556000 | 101576000 | 101556000 | 101576000 | 0 | 0 |
| 101556000 | 101576000 | 101556000 | 101576000 | 0 | 0 |
| 101558000 | 101568000 | 101558000 | 101568000 | 0 | 0 |
| 101558000 | 101568000 | 101558000 | 101567997 | 0 | -3 |
| 101560000 | 101565000 | 101559999 | 101564999 | -1 | -1 |
| 101561000 | 101562000 | 101561000 | 101562000 | 0 | 0 |
|  |  |  |  |  |  |
| **MATCHCLIP** |  |  |  |  |  |
| 101545220 | 101630000 | 101545220 | 101630001 | 0 | 1 |
| 101555000 | 101605000 | 101555000 | 101604999 | 0 | -1 |
| 101556000 | 101576000 | 101556000 | 101576001 | 0 | 1 |
| 101556000 | 101576000 | 101556001 | 101576000 | 1 | 0 |
| 101556000 | 101576000 | 101556001 | 101576000 | 1 | 0 |
| 101556000 | 101576000 | 101556001 | 101576004 | 1 | 4 |
| 101558000 | 101568000 | 101558001 | 101568000 | 1 | 0 |
| 101560000 | 101565000 | 101559997 | 101564998 | -3 | -2 |
| 101560000 | 101565000 | 101559999 | 101565000 | -1 | 0 |
| 101560000 | 101565000 | 101559999 | 101565000 | -1 | 0 |
| 101560000 | 101565000 | 101560000 | 101565000 | 0 | 0 |
| 101561000 | 101562000 | 101561000 | 101562001 | 0 | 1 |
|  |  |  |  |  |  |
| **SOFTSEARCH** |  |  |  |  |  |
| 101545220 | 101630000 | 101544746 | 101630188 | -474 | 188 |
| 101545220 | 101630000 | 101544966 | 101630372 | -254 | 372 |
| 101545220 | 101630000 | 101545220 | 101630208 | 0 | 208 |
| 101545220 | 101630000 | 101545220 | 101630236 | 0 | 236 |
| 101545220 | 101630000 | 101545221 | 101630131 | 1 | 131 |
| 101545220 | 101630000 | 101545221 | 101630166 | 1 | 166 |
| 101545220 | 101630000 | 101545221 | 101630173 | 1 | 173 |
| 101545220 | 101630000 | 101545221 | 101630184 | 1 | 184 |
| 101545220 | 101630000 | 101545221 | 101630189 | 1 | 189 |
| 101545220 | 101630000 | 101545221 | 101630203 | 1 | 203 |
| 101545220 | 101630000 | 101545221 | 101630244 | 1 | 244 |
| 101556000 | 101576000 | 101555640 | 101576323 | -360 | 323 |
| 101556000 | 101576000 | 101555747 | 101576419 | -253 | 419 |
| 101556000 | 101576000 | 101556002 | 101576163 | 2 | 163 |
| 101556000 | 101576000 | 101556004 | 101576105 | 4 | 105 |
| 101556000 | 101576000 | 101556004 | 101576177 | 4 | 177 |
| 101556000 | 101576000 | 101556005 | 101576139 | 5 | 139 |
| 101556000 | 101576000 | 101556005 | 101576157 | 5 | 157 |
| 101556000 | 101576000 | 101556005 | 101576159 | 5 | 159 |
| 101556000 | 101576000 | 101556005 | 101576178 | 5 | 178 |
| 101556000 | 101576000 | 101556005 | 101576206 | 5 | 206 |
| 101556000 | 101576000 | 101556005 | 101576244 | 5 | 244 |
| 101556000 | 101576000 | 101556005 | 101576277 | 5 | 277 |
| 101560000 | 101565000 | 101559460 | 101564998 | -540 | -2 |
| 101560000 | 101565000 | 101559999 | 101565188 | -1 | 188 |
| 101560000 | 101565000 | 101560000 | 101565132 | 0 | 132 |
| 101560000 | 101565000 | 101560001 | 101565128 | 1 | 128 |
| 101560000 | 101565000 | 101560001 | 101565149 | 1 | 149 |
| 101560000 | 101565000 | 101560001 | 101565153 | 1 | 153 |
| 101560000 | 101565000 | 101560001 | 101565158 | 1 | 158 |
| 101560000 | 101565000 | 101560001 | 101565172 | 1 | 172 |
| 101560000 | 101565000 | 101560001 | 101565191 | 1 | 191 |
| 101560000 | 101565000 | 101560001 | 101565214 | 1 | 214 |
| 101560000 | 101565000 | 101560001 | 101565365 | 1 | 365 |
| 101561000 | 101562000 | 101561001 | 101562116 | 1 | 116 |
| 101561000 | 101562000 | 101561001 | 101562126 | 1 | 126 |
| 101561000 | 101562000 | 101561001 | 101562144 | 1 | 144 |
| 101561000 | 101562000 | 101561001 | 101562182 | 1 | 182 |
| 101561000 | 101562000 | 101561001 | 101562184 | 1 | 184 |
| 101561000 | 101562000 | 101561001 | 101562197 | 1 | 197 |
| 101561000 | 101562000 | 101561001 | 101562215 | 1 | 215 |
| 101561000 | 101562000 | 101561001 | 101562220 | 1 | 220 |
| 101561000 | 101562000 | 101561001 | 101562290 | 1 | 290 |
| 101561000 | 101562000 | 101561001 | 101562328 | 1 | 328 |
| 101555000 | 101605000 | 101554996 | 101604294 | -4 | -706 |
| 101555000 | 101605000 | 101554996 | 101604298 | -4 | -702 |
| 101555000 | 101605000 | 101554996 | 101604360 | -4 | -640 |
| 101555000 | 101605000 | 101554998 | 101604296 | -2 | -704 |
| 101555000 | 101605000 | 101554998 | 101604318 | -2 | -682 |
| 101555000 | 101605000 | 101554998 | 101604333 | -2 | -667 |
| 101555000 | 101605000 | 101555165 | 101604503 | 165 | -497 |
| 101555000 | 101605000 | 101555185 | 101604481 | 185 | -519 |
| 101555000 | 101605000 | 101555205 | 101604545 | 205 | -455 |
| 101555000 | 101605000 | 101555299 | 101604592 | 299 | -408 |
| 101555000 | 101605000 | 101555496 | 101604866 | 496 | -134 |
| 101556000 | 101576000 | 101555995 | 101575372 | -5 | -628 |
| 101556000 | 101576000 | 101555999 | 101575288 | -1 | -712 |
| 101556000 | 101576000 | 101555999 | 101575293 | -1 | -707 |
| 101556000 | 101576000 | 101555999 | 101575322 | -1 | -678 |
| 101556000 | 101576000 | 101555999 | 101575353 | -1 | -647 |
| 101556000 | 101576000 | 101555999 | 101575357 | -1 | -643 |
| 101556000 | 101576000 | 101555999 | 101575387 | -1 | -613 |
| 101556000 | 101576000 | 101556330 | 101575628 | 330 | -372 |
| 101556000 | 101576000 | 101556435 | 101575760 | 435 | -240 |
| 101556000 | 101576000 | 101556435 | 101575811 | 435 | -189 |
| 101556000 | 101576000 | 101556483 | 101575826 | 483 | -174 |
| 101556000 | 101576000 | 101556522 | 101575925 | 522 | -75 |
| 101558000 | 101568000 | 101557999 | 101567203 | -1 | -797 |
| 101558000 | 101568000 | 101557999 | 101567318 | -1 | -682 |
| 101558000 | 101568000 | 101557999 | 101567324 | -1 | -676 |
| 101558000 | 101568000 | 101557999 | 101567325 | -1 | -675 |
| 101558000 | 101568000 | 101557999 | 101567342 | -1 | -658 |
| 101558000 | 101568000 | 101557999 | 101567381 | -1 | -619 |
| 101558000 | 101568000 | 101557999 | 101567382 | -1 | -618 |
