# Supplementary material for: SRBreak: A Read-Depth and Split-Read Framework to Identify Breakpoints of Different Events Inside Simple Copy-Number Variable Regions
Source: Front Genet. 2016 Sep 15;7:160. doi: 10.3389/fgene.2016.00160 (PMC5023681; doi:10.3389/fgene.2016.00160)
Supplement: TABLE S5 — The results of the 1000 bp event called by SRBreak for 15 duplicated samples. The best results were seen for window sizes of 500 bp: the package called exactly 13/15 duplication samples [failed to called samples of low (1–2x) coverage]. [file Table_5.DOCX]

**Table S5**

| Window | Paired-end read | Single-end read |
| --- | --- | --- |
| 50 | 0 | 0 |
| 100 | 11 | 3 |
| 250 | 12 | 12 |
| 500 | 13 | 12 |
| 1000 | 0 | 0 |
