# Supplementary material for: SRBreak: A Read-Depth and Split-Read Framework to Identify Breakpoints of Different Events Inside Simple Copy-Number Variable Regions
Source: Front Genet. 2016 Sep 15;7:160. doi: 10.3389/fgene.2016.00160 (PMC5023681; doi:10.3389/fgene.2016.00160)
Supplement: TABLE S6 — Concordant results between SRBreak and McCarroll et al. (2008) and The 1000 Genomes Project (2012) for three loci NEGR1, LCE3, and IRGM with different mapping qualities (QUAL). These results were obtained by running SRBreak with a mappability file and removing reads having QUALs lower than 1, 10, or 20 as described in columns. [file Table_6.DOCX]

**S6 Table**

|  | QUAL = 1 | QUAL = 10 | QUAL = 20 |
| --- | --- | --- | --- |
| NEGR1 | 0.74 | 0.76 | 0.78 |
| LCE3 | 1.00 | 1.00 | 0.99 |
| IRGM | 0.81 | 0.78 | 0.39 |
